# Hierarchical Leak Detection and Localization Method in Natural Gas Pipeline Monitoring Sensor Networks

**DOI:** 10.3390/s120100189

**Published:** 2011-12-27

**Authors:** Jiangwen Wan, Yang Yu, Yinfeng Wu, Renjian Feng, Ning Yu

**Affiliations:** School of Instrumentation Science and Opto-Electronics Engineering, Beijing University of Aeronautics and Astronautics, Beijing 100191, China; E-Mails: jwwan@buaa.edu.cn (J.W.); yfwu@buaa.edu.cn (Y.W.); rjfeng@buaa.edu.cn (R.F.) nyu@buaa.edu.cn (N.Y.)

**Keywords:** leak detection, leak point localization, hierarchical, pipeline monitoring, sensor networks

## Abstract

In light of the problems of low recognition efficiency, high false rates and poor localization accuracy in traditional pipeline security detection technology, this paper proposes a type of hierarchical leak detection and localization method for use in natural gas pipeline monitoring sensor networks. In the signal preprocessing phase, original monitoring signals are dealt with by wavelet transform technology to extract the single mode signals as well as characteristic parameters. In the initial recognition phase, a multi-classifier model based on SVM is constructed and characteristic parameters are sent as input vectors to the multi-classifier for initial recognition. In the final decision phase, an improved evidence combination rule is designed to integrate initial recognition results for final decisions. Furthermore, a weighted average localization algorithm based on time difference of arrival is introduced for determining the leak point’s position. Experimental results illustrate that this hierarchical pipeline leak detection and localization method could effectively improve the accuracy of the leak point localization and reduce the undetected rate as well as false alarm rate.

## 1. Introduction

In the present age, natural gas has become one of the most important energy resources in the World, which primarily depends on the pipeline transportation. However, with the widespread demand for natural gas supplies, the pipeline leak detection problem has become increasingly prominent [1,2]. Conventional leak detection methods mainly rely on periodical inspections conducted by maintenance personnel, which needs intensive human involvement, but periodical inspection does not provide real time monitoring of the pipeline. Accordingly, a leak may not be detected in time and this may bring about a great deal of economic losses and environmental pollution. Real time pipeline monitoring systems based on wired or wireless sensors have been studied in [3–5]. The wire-based techniques connect the sensors along the pipelines with wires. Monitoring information measured by each sensor is transmitted to the monitoring control center through these wires. Nevertheless, the wire-based monitoring systems are vulnerable to damage in any part of the networks and the deployment cost in an underground scenario is very expensive. Wireless sensor networks (WSNs) [6,7], on the other hand, are a much more robust and efficient option to monitor pipelines. Since 2006, many studies have been carried out on WSNs for pipeline monitoring. Jawhar *et al.* [8] proposed a framework for pipeline infrastructure monitoring using WSNs and designed a routing protocol for transmitting the information from sensor nodes to the sink. Guo *et al.* [9] put forward a distributed sensing data propagation algorithm based on graded residual energy used in pipeline monitoring sensor networks. The algorithm can achieve balanced energy consumption among sensor nodes and prolong the network lifetime. Ivan *et al.* [10] described a system called PipeNet used for detecting and localizing leaks and failures in water transmission pipelines. The system is composed of a host of sensor nodes with perception, computation and wireless communication capacities by self-organization. According to the acoustic signals, velocity (flow) signals and pressure transient signals captured by nodes, the system can diagnose the current state of the pipeline more accurately [10].

Although some monitoring systems based on WSNs have been developed in the last several years, research on information processing in this type of networks is relatively scarce. Yu *et al.* [11] analyzed the features of monitoring information in pipeline networks in detail. Imad *et al.* [12] presented a model of in-network information processing for pipeline monitoring based on WSNs, but did not give any specific algorithms. This is mainly because monitoring information shows uncertainty and diversity in expressive form, huge amounts in numbers and complicated relationships [11]. Consequently, so far there has not been a kind of method which can effectively process monitoring information of natural gas pipelines. This mainly reflects the following aspects [13,14]: in the first place, under urban area circumstances, sensors may be affected by background noise so that detection information has great ambiguity. This is due to the fact that noise may submerge the useful signals, which will result in a false diagnosis by the monitoring system. In addition, the information provided by single-type sensors is always incomplete or inaccurate as a result of their own deficiencies and environmental interference, so each node may integrate several different types of sensors, such as acoustic sensor, flow sensor and pressure sensor and so on. Thus we need to design a reliable method for multi-source information processing. Moreover, to avoid the monitoring blind zone, when a node is out of action, the detection range among nodes should have a certain degree of overlap. In this case, multiple nodes detect the pipeline leak simultaneously when a leak occurs, but affected by the monitoring position, different nodes may generate conflicting diagnosis results, which make it difficult for the monitoring system to make a right decision, so we need to devise a conflicting results solution method for making a reliable decision according to the diagnosis results from different nodes, avoiding the false alarm problem.

In view of the issues above, in this paper we analyze the benefits and drawbacks of existing methods and propose a novel hierarchical pipeline leak detection and localization method. At different levels, we adopt different detection methods to process leak information. The effectiveness of the new method is verified by simulation experiments. Our results demonstrate that the hierarchical method can detect pipeline leaks and localize leak points accurately and effectively.

The rest of the paper is organized as follows: in Section 2, we review some of existing methods for pipeline leak detection and localization. The preliminaries, including the architecture of pipeline monitoring sensor networks and hierarchical leak detection and localization model, are briefly illustrated in Section 3. Section 4 describes the algorithm in detail. Section 5 evaluates the performance of hierarchical leak detection and localization method through simulation experiments. Finally, Section 6 concludes this paper.

## 2. Existing Leak Detection and Localization Methods

Existing pipeline leak detection and localization methods can be divided into three categories based on signal processing, model estimation and knowledge.

### 2.1. Methods Based on Signal Processing

#### 2.1.1. Negative Pressure Wave

When a leak happens along the pipeline, the liquid density of the leak point declines immediately due to the fluid medium losses and the pressure drops. Then the pressure wave source spreads out from the leak point to the leak upstream and downstream ends. Taking the pressure before the leak as the reference criterion, the wave generated by such a leak is called the negative pressure wave. When the negative pressure wave reaches the pipeline terminal end, it will cause the drop of first the station inlet pressure and then the station outlet pressure. The pressure reduction signal is collected by pressure sensors installed on both ends of the pipeline. As the locations of the leak are different, different time difference of the negative pressure wave are captured. Based on the different time difference that pressure sensors on both sides detect, pipeline length and negative pressure wave velocity, the leak point can be determined [15,16]. The negative pressure wave method is not dependent on system hardware and it also does not need to set up the mathematical model, which means less calculation costs. However, it requires that the leak be quick and abrupt, since especially in the case of a slow leak, there is no obvious negative pressure wave.

#### 2.1.2. Mass Balance

The working principle of mass balance method for leak detection is straightforward [17,18]: if a leak occurs, the mass balance equation (accounting for the input and output mass flow rates and also the line pack variable rate) presents a systematic deviation. Although simple and certainly reliable, the primary difficulty in implementing this principle in practice derives from the huge variations experienced by the line pack term. This effect implies a very long detection time and, therefore, a frequently unacceptable leaked mass before an alarm is declared. Although the principle of mass balance method is simple, this method is very sensitive to arbitrary disturbances and dynamics of the pipeline, which may lead to false detection issues.

#### 2.1.3. Pressure Point Analysis

Pressure point analysis (PPA) is one of the more popular system diagnosis methods that are available [19]. The PPA method detects leaks by monitoring pipeline pressure at a single point along the line and comparing it with a running statistical trend constructed from previous pressure measurements. The combination of selective filtering and software thresholds determines whether the behavior of successive measurements contains evidence of a leak. The PPA method just requires pressure signals from one or several detection points, so the advantages of this method are cost-effectiveness and easy maintenance. Additionally, PPA can detect small leaks which cannot be detected by other methods. However, it is difficult for this method to localize leak points. Therefore, the application range of PPA is too narrow.

#### 2.1.4. Acoustic Correlation Analysis

This method adopts the sound generated by pipeline leak as signal source, installs acoustic sensors along the pipeline to detect sound in leak point and uses signal processing technology for leak detection and localization [20,21]. Due to the limitations of object detection and the application environment, it is absolutely necessary to deploy many acoustic sensors along the pipeline for long distance detection. This method has the advantages of high detection and localization accuracy. Especially, this method does not need to set up complex mathematical model and could detect small leaks.

#### 2.1.5. Spectral Analysis Response

The principle of spectral analysis response for pipeline leak detection is as follows: when a mini-leak occurs along the pipeline, it will generate a leak signal such as an ultrasonic wave. By extracting the characteristic parameters of leak signals for further analysis, we could obtain the corresponding relationship between ultrasonic spectrum and leak parameters such as aperture of leak point and pipeline inner pressure to judge the state and tendency of the leak point, thereby significantly enhancing the accuracy of the leak detection. Generally, spectral analysis response methods are based on Fast Fourier Transform (FFT) [22,23]. The accuracy of spectral analysis mainly depends on the eigenvalue problem, which can be solved by a filter diagonalization method (FDM) [24,25].

### 2.2. Methods Based on Model Estimation

#### 2.2.1. State Observer

The working principle of a state observer for pipeline leak detection is as follows: the state equations of pressure and flow amount are set up. Two pressure extremes are detected as inputs, proper algorithms are adopted for leak point detection and localization according to the error signal from measured and observed values. However, this method supposes that both pressure extremes are not affected by the size of the leak, so it is only suitable for the case of small leaks.

#### 2.2.2. System Identification

This method can effectively distinguish the pipeline model and judge whether a leak happens by comparing it with pipeline actual value. In the case of pipeline integrity, both a fault sensitive model and a fault free model are built. For the fault sensitive model, correlation analysis is used for leak detection. For the fault free model, proper algorithms are introduced for leak point localization. Like the state observer method, this method supposes that both pressure extremes are not affected by the magnitude of the leak. Apart from that, it also needs to exert an M-sequence stimulus signal on the pipeline.

#### 2.2.3. Kalman Filter [26]

This method first sets up discrete state space models of pressure and flow. Pressure and flow at the head and end of the pipeline are used as inputs. The whole pipeline is divided into several sections and three sorts of states (pressure, flow and leak amount) are set at each section point. Then an appropriate discriminate criterion is adopted for leak point detection and localization. However, this method has to acquire mean value and variance of process noise in advance and the degrees of accuracy of detection and localization are related to the number of sections.

#### 2.2.4. Impedance Method [27]

The principle of the impedance method for leak detection can be given as follows: the hydraulic impedance is defined as the ratio of the head fluctuation to the discharge and the characteristic impedance is defined according to the pipe parameters and propagation constant. When the pipe friction is neglected, the final formulation of the pipe impedance with a leak is a function of hydraulic impedance. Therefore, the leak point position is determined by calculating the value of hydraulic impedance for each point where a leak could be detected. The impedance method is mainly used for linear pipelines with less curves and bends, because complicated pipelines with many curves bring difficulties and major uncertainties in the calculation of characteristic impedance and in the statement of boundary conditions.

#### 2.2.5. Standing Wave Difference Method

Standing wave difference method (SWDM) is a promising leak detection method for water pipeline transmission systems, which is based on the technology used in electrical engineering to determine leak point position [28]. Its principle is that SWDM utilizes sinusoidal excitation of the pipeline at one end using an oscillator and simultaneous measurement of head and discharge. Every discontinuity of the pipe impedance (*i.e.*, the ratio between head fluctuation and discharge) reflects incident waves, creating residual standing waves. The distance between the excitation site and pipeline discontinuity is determined by the analysis of the respective resonance frequencies.

### 2.3. Methods Based on Knowledge

#### 2.3.1. Support Vector Machine

The method based on support vector machine (SVM) for leak detection and localization is to monitor the parameters at a number of sensor nodes in the pipeline networks under consideration and to feed these parameters’ values into a SVM trained to predict leak size and leak location [29,30]. The SVM is trained on a number of samples representing the presence of leaks of various sizes and locations in the pipeline monitoring networks.

#### 2.3.2. Pattern Recognition

The detection principle of the pattern recognition method is based on transient feature extraction and structural pattern recognition for the negative pressure wave caused by a pipeline leak [31,32]. Since it is quite different from that caused by adjusting pumps and valves, describing the negative pressure wave by pattern recognition method and building classification system of wave structure are able to effectively distinguish between normal adjustment states and leak states. This type of detection method can effectively reduce the false alarm rate and the missing detection rate, and improve the accuracy of leak detection.

#### 2.3.3. Expert System

A leak diagnosis expert system based on leak mechanism is a type of complex nonlinear distributed parameter control system [33,34]. The whole reasoning process is how to synthetically utilize experience knowledge, leak pattern, leak mechanism model and physics laws. The system first completes the breadth search for the most serious abnormal phenomenon of the pipeline. Then it carries out the depth-first search, accomplishing effective qualitative diagnosis based on experience knowledge to determine whether a leak happens.

## 3. Network Architecture and Hierarchical Model for Leak Detection and Localization

### 3.1. Architecture of Pipeline Monitoring Sensor Networks

**Figure 1.**
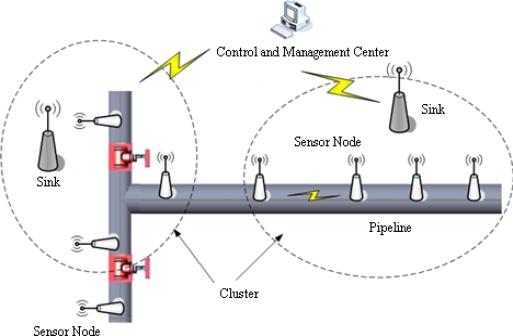
Architecture of pipeline monitoring sensor networks.

Natural gas pipeline monitoring sensor networks are made up of a great number of sensor nodes, the sinks and a control and management center. The whole networks adopt a sort of clustering structure, which is described as Figure 1. The sensor nodes, installed along the pipeline, are in charge of signal collecting and data preprocessing. Then the processing results are sent to the sink via a multi-hop route. The monitoring networks take an in-tube communication mode for message exchange between sensor nodes. The sink, as the cluster leader, is responsible for managing sensor nodes in its cluster, combining preprocessing results for final decision and localizing leak position if a leak has occurred. Finally, the diagnosis result is sent to the control and management center, which judges whether a leak warning alarm should be announced.

### 3.2. Hierarchical Leak Detection and Localization Model

According to the architecture of monitoring networks and the monitoring information features, we set up a hierarchical model for leak detection and localization as follows: in the first place, the original leak signals are easily disturbed by background noise and transmission route, which may lead to the ambiguity of detection information. In order to diminish the disturbance impact and improve the detection precision, wavelet transform technology is introduced to cope with original leak signals and the leak characteristic parameters are extracted as well. Secondly, SVM multiple classifiers are employed to integrate the leak characteristic parameters in monitoring networks. This kind of classifiers not only could enhance the ability of anti-interference, but also could accurately recognize the current state of a pipeline. Thirdly, due to the influence of monitoring position, sensor nodes in different positions may generate conflicting results, which makes it difficult for the monitoring networks to provide a correct decision. To solve this problem, a recognition result from a single node, which is regarded as the independent evidence, will be combined with results from other nodes at the sink to increase the focal degree of the correct proposition. Furthermore, if there has been a leak along the pipeline, the position of the leak point can be precisely determined by the localization results among nodes.

**Figure 2.**
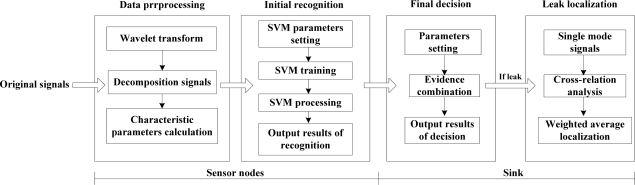
Hierarchical model for leak detection and localization.

Based on the analysis above, the process of leak detection and localization is divided into three parts, shown as Figure 2. At sensor nodes, we use wavelet transform technology to deal with original leak signals as well as obtain characteristic parameters of the signals in both the time and frequency domains. Then, we build the model of SVM multiple classifiers and acquire the recognition results of each node. At the sink, we design an improved evidence combination rule based on the degree of reliability for the final decision making from the recognition results of several nodes, and judge the state of the pipeline. If the state corresponds to a leak, a weighted average localization algorithm based on time difference of arrival (TDOA) is introduced to precisely calculate the position of the leak point. The whole model adopts a hierarchical configuration, in which the result from the former level is regarded as the input of the next level. Thus the model is able to separately achieve multiple different levels of leak information processing, which ensures the accuracy and reliability of the diagnosis results.

## 4. Algorithm Design

### 4.1. Leak Detection Algorithm

#### 4.1.1. Leak Information Preprocessing Based on Wavelet Transformation

##### (1) Selection of Wavelet Basis

In this study, wavelet in series db is adopted to transform the original signals. This sort of wavelet has the following advantages: (i) it is sensitive to the leak signals and can highlight the singular characteristics of signals; (ii) it can reduce the signal distortion and ensure perfect signal reconstruction as well as good performance of time-frequency analysis.

##### (2) Determination of Decomposition Scale

We make the discrete wavelet transform for leak signals with background noise. Generally, the larger the decomposition scale is, the more favorable it is to eliminate the noise [35]. However, some important local singular characteristics may be lost if the decomposition scale is too large. According to the frequency characteristics of detection signals, we suppose that signal sampling frequency is *f_s_* and the lowest identification frequency is *f_min_*. Thus the maximum decomposition scale *n* should satisfy the following relationship expression:

(1) $$\frac{f_{s}}{2^{n+1}}\geq f_{\text{min}}$$

##### (3) Wavelet Denoising Method of Threshold Selection

After calculating the maximum decomposition scale *n*, we decompose the leak signals into *n* levels. Considering that wavelet spectrums of leak signals and noise signals have different performance characteristics in every scale, we select an appropriate threshold by a heuristic method. For the points whose value is less than the threshold value, its coefficient is set to 0, while for the points whose value is more than the threshold value, its coefficient is set to the difference between the point value and the threshold value. Then we reconstruct the decomposed signals and obtain the single mode signals without noise.

##### (4) Characteristic Parameters Calculation

We carry out the time domain analysis for single mode signals and extract the characteristic parameters which can completely reflect pipeline leak, such as peak value, average amplitude, variance, root amplitude and so on.

#### 4.1.2. Leak Initial Recognition Based on SVM Multiple Classifiers

**Figure 3.**
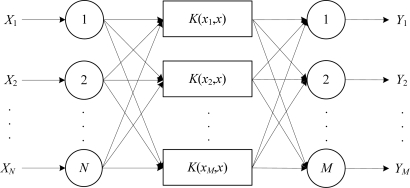
Structure of SVM multiple classifiers.

At sensor nodes, the leak recognition model is built based on SVM multiple classifiers because the SVM method is able to detect small leaks and slow leaks which could not be detected by the traditional methods. Also, the SVM method avoids the large sample requirements of traditional classification methods [36,37]. To build a model of SVM multiple classifiers includes designing the following aspects: input vector, classification principle, inner product function, penalty coefficient. Sensor nodes in the networks adopt the same structure of SVM multiple classifiers, shown in Figure 3. Where *X_i_* (*i* = 1,2,…,*N*) denote the input characteristic parameters, and *Y_j_* (*j* = 1,2,…,*M*) denote the output results from SVM multiple classifiers.

##### (1) Selection of Input and Output Variables

Related studies have demonstrated that (i) peak value, (ii) average amplitude, (iii) variance and (iv) mean square root are parameters which can reflect the fluctuation of stress waves caused by pipeline leaks; (v) peak factor, (vi) pulse factor and (vii) margin factor are parameters which can reflect the impact of dramatic changes of stress waves; (viii) skewness factor and (ix) kurtosis factor are parameters which can reflect the changes of amplitude distribution of stress waves [38]. Consequently, the nine parameters mentioned above are selected as characteristic input vectors of the SVM multiple classifiers.

Owing to the fact that a single SVM only resolves the two-classifier problem, we set up a SVM model composed by several classifiers to distinguish different states of the pipeline. According to the application requirements analysis for leak detection, the pipeline has three types of recognition states: normal, small leak and big leak. As a result, we need to construct three SVMs to correspond with the three kinds of states. For the *i*th SVM, the recognition output of *i*th pipeline state is +1 while the outputs of other two states are both −1. Namely, the number of output variables is 3. When the pipeline states are normal, small leak and big leak, output variables of multiple classifiers are (+1, −1, −1), (−1, +1, −1) and (−1, −1, +1), respectively.

##### (2) Classification Principle and Parameters Optimization

The classification principle of SVM multiple classifiers is based on structural risk minimization principle and kernel function method [39]. The expression of discriminant function is:

(2) $$f(x)=\text{sgn}(\sum_{i=1}^{n}\alpha_{i}y_{i}k(x_{i},x)+b)$$

where *x_i_* and *x* represent training and measured samples, respectively; *y* = {1, +1} is the classification label. *α* denotes Lagrange coefficient vector, which satisfies that 0 ≤ *α_i_* ≤ *C* and $\sum_{i=1}^{n}a_{i}y_{i}=0$, *i* = 1,2,…,*n*, where *C* is the penalty factor. In Equation (2), *b* denotes the classification threshold value and *k*(*x_i_*,*x*) is the kernel function. In this paper RBF kernel function is adopted and its expression is:

(3) $$k(x_{i},x)=\text{exp}[-\gamma {\left\|x_{i}-x\right\|}^{2}]$$

where *γ* is the parameter of the kernel function. Simulation experiments show that RBF kernel function has better classification results than others.

In the aspect of parameter selection, both *C* and *γ* are important parameters, which are able to directly affect classification precision. Here, a bilinear search method is used to work out the optimal parameters. Its principle is that different values of parameters combinations (*C*, *γ*) have different properties to correspond with, so we use log*C* and log*γ* as the coordinates of parameter space and parameter combination (*C*, *γ*) for maximum learning precision will occur in the vicinity of the straight line log *γ* = log *C* – log *C̃*. The specific algorithm is as follows: (i) we calculate the optimal *C̃* for maximum learning precision from linear SVM; (ii) for the SVM with RBF kernel function, we fix *C̃*. If *C̃* satisfies log *γ* = log *C* – log *C̃*, we train the SVM for obtaining the optimal parameter values.

##### (3) Probability Output of SVM Multiple Classifiers

The standard SVM output is a hard decision output so that it is likely to cause excessive dependence on the voting method [40]. However, it needs a SVM with soft decision output in practical application. Therefore, in order to get posterior probability output, we utilize a sigmoid function to map the SVM output *f*(*x*) to the interval (0, 1). The expression of posterior probability output is:

(4) $$P(y=1|f(x))=\frac{1}{1+\text{exp}(Gf(x)+H)}$$

where the parameters *G* and *H* could be obtained from the following Equation:

(5) $$\text{min}-(\sum_{i}t_{i}\;\text{log}(p_{i})+(1-t_{i})\text{log}(1-p_{i}))$$

where $p_{i}=\frac{1}{1+\text{exp}(Gf(x_{i})+H)}$, $t_{i}=\frac{y_{i}+1}{2}$ and *y_i_* is the classification label of sample *i*.

#### 4.1.3. Final Decision Based on Improved Evidence Combination Rule

According to the practical output requirement of pipeline leak detection, we set up the decision model based on an improved evidence combination rule.

##### (1) Frames of Discernment

Let *θ* be the frame of discernment, containing *N* exclusive and exhaustive hypotheses, and let 2*^θ^* denote its power set. In this paper, frames of discernment in pipeline monitoring sensor networks are *θ* = {*A*_1_, *A*_2_, *A*_3_}, where *A*_1_, *A*_2_ and *A*_3_ respectively represent the normal, small leak and big leak states.

##### (2) Basic Probability Assignment (BPA)

A basic probability assignment is a function *m* from 2*^θ^* to [0, 1] verifying:

(6) $$\{\begin{array}{l} m(\Phi )=0\\ \sum_{A\subseteq \theta }m(A)=1 \end{array}$$

For any *A*, *m*(*A*) represents the belief that one is willing to commit to *A*, given a certain piece of evidence. Therefore, how to assign the values of *m*(*A_i_*) is the main problem to be dealt with. Here we propose the concept of error upper bound in SVM classification recognition: if a set of training samples could be divided by an optimal classification surface, the upper bound of classification error rate of testing samples is the ratio of average support vector number of training samples to total number of training samples. That is:

(7) $$E(P(\mathrm{error}))\leq \frac{E_{\mathrm{sv}}}{E_{t}-1}$$

where *E_sv_* and *E_t_* respectively denote the average support vector number and total number of training samples. Equation (7) precisely reflects the uncertainty of sample *x* by the SVM method, which corresponds to *Θ* in frames of discernment. As a consequence, we employ the following method to assign BPA values of *A_i_* (*i* = 1,2,…,*N*).

For the *i*th point in the sample set, the probability output of initial recognition results could be calculated by trained SVM multiple classifiers. The BPA value corresponding to *A_j_* in initial recognition results is:

(8) $$\begin{array}{lll} m_{i}(A_{j}) & = & \frac{1}{1+\text{exp}(\mathrm{Gf}(x_{j})+H)}\;\frac{s_{j}}{l-1}+\frac{1}{k-1}\\ & & \left[\sum_{m=1,m\neq j}^{3}\;\frac{\text{exp}(\mathrm{Gf}(x_{j})+H)}{1+\text{exp}(\mathrm{Gf}(x_{j})+H)}\;\frac{s_{m}}{l-1}\right]\\ m_{i}(\Theta ) & = & \frac{1}{k-1}\;(\sum_{m=1}^{3}\;\frac{\text{exp}(\mathrm{Gf}(x_{j})+H)}{1+\text{exp}(\mathrm{Gf}(x_{j})+H)}\;\frac{s_{m}}{l-1}) \end{array}$$

where $\frac{1}{1+\text{exp}(Gf(x_{j})+H)}$ is the posterior probability output of *j*th two-classification SVM and it could be calculated by Equation (4). The term *s_j_* denotes the number of support vector corresponding to the *j*th SVM. *l* is the total number of training samples. *Θ* denotes the uncertainty of recognition result. The normalization result of *m*(*A*) is shown as follows:

(9) $$m_{i}^{\prime }\;(A_{j})=m_{i}\;(A_{j})/\sum_{j=1}^{k}m_{i}\;(A_{j})$$

In this way, we can get the BPA function *m*′*_i_* corresponding to *i*th test sample point. In the practical process of pipeline leak detection, the closer the sensor node is to the leak point, the more reliable the detection result is. In contrast, the result is less reliable when the sensor node is farther from the leak point as a result of more interference in leak signals. In order to reduce the effect of the unreliable evidence on combination result and promote the degree of focus of the correct proposition, the BPA function values produced as outputs from SVM multiple classifiers need to be preprocessed before evidence combination. Here the preprocessing method is based on node reliability degree: we suppose the distance between the *i*th node and the leak point is *d_i_* and the degree of reliability of the node closest to the leak point is 1. The reliability degrees of other nodes are given as follows:

(10) $$\{\begin{array}{l} d_{\text{min}}=\text{min}(d_{i})\\ {\mathrm{Cred}}_{i}=\sqrt[{p}]{d_{\text{min}}/d_{i}} \end{array}$$

where *p* is the impact factor of reliability degree. After a great deal of experimental research, *p* = 8 in this paper. In order to meet the requirement of the sum of BPA function of each proposition is 1, we process the BPA function via reliability degree expression:

(11) $$\{\begin{array}{l} m_{i}^{*}\;(A_{j})={\mathrm{Cred}}_{i}m_{i}^{\prime }\;(A_{j}),\;\;\;A_{j}\neq \emptyset \\ m_{i}^{*}\;(\Theta )=1-\sum_{A_{j}\subset \Theta }m_{i}^{*}(A_{j}) \end{array}$$

##### (3) Evidence Combination

Suppose *m*_1_ and *m*_2_ respectively represent the BPA function of the same frame of discernment, focal elements of which are *A_i_* and *B_j_*. The combination rule of *A* and *B* are shown:

(12) $$\{\begin{array}{l} m_{1⊕2}\;(A)=0,\;A=\emptyset \\ m_{1⊕2}\;(A)=\frac{\sum_{A_{i}\cap A_{j}=A}m_{1}(A_{i})m_{2}(B_{j})}{1-K} \end{array}$$

where $K=\sum_{A_{i}\cap A_{j}=\phi }m_{1}(A_{i})m_{2}\;(B_{j})$ denotes the conflict weight.

##### (4) Decision Making Based on Maximum Trust Degree

According to evidence combination results from Equation (12), we adopt maximum trust degree method to make final decision.

∃*A*_1_, *A*_2_ ⊂ *θ*, *m*(*A*_1_) = max{*m*(*A_k_*), *A_k_* ⊂ *θ*} and *m*(*A*_2_) = max{*m*(*A_k_*), *A_k_* ⊂ *θ*, 且*A_k_* ≠ *A*_1_}. If the following relationship expression is satisfied:

(13) $$\{\begin{array}{c} m(A_{1})-m(A_{2})>\varepsilon_{1}\\ m(\Theta )<\varepsilon_{2}\\ m(A_{1})>m(\Theta ) \end{array}$$

*A*_1_ is the decision result, where, *ε*_1_ and *ε*_2_ are threshold values. In this paper *ε*_1_ = 0.15 and *ε*_2_ = 0.1.

### 4.2. Leak Point Localization Algorithm

#### 4.2.1. Principle of Time Difference of Arrival

**Figure 4.**
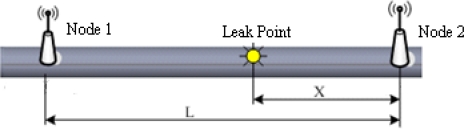
Principle of TDOA localization.

The localization method based on time difference of arrival (TDOA) is described as follows [41]: Several sensor nodes are placed at fixed points to make up the sensor array in accordance with some geometric relationship. These nodes measure and calculate the relative time difference of signal waves transmitting to each sensor node. Then time differences are substituted into the equations which satisfy the geometric relationship of sensor array for solving the leak point coordinate. Furthermore, leak signals will lose some energy during the process of propagation, that is to say, signal intensity will decline with the increase of the distance between leak point and sensor position. According to this feature, the leak source region can be roughly determined via the signal intensity received by sensor nodes and leak points can be accurately localized by analyzing the signal attenuation characteristics. Figure 4 shows the principle of TDOA localization through two sensor nodes. The location Equation of leak point is:

(14) $$X=\frac{L-\alpha \Delta t}{2}$$

where *X* denotes the distance between the leak point and the nearest node. Because all the nodes in the monitoring networks have definite location coordinates, the location of the leak point could be determined according to *X*. *L* is the distance between upstream and downstream nodes, the value of which is certain when the networks are deployed. *α* denotes the wave propagation speed and the value of *α* is able to be obtained from engineering handbook or practical measurement. Δ*t* denotes time difference of leak signal arriving at upstream and downstream nodes, which is the key problem of time of arrival localization.

#### 4.2.2. Process of Leak Point Localization

Leak point localization is accomplished at the sink. The localization process is divided into three parts: signals grouping, determination of time difference and weighted average localization.

##### (1) Signals Grouping

**Figure 5.**
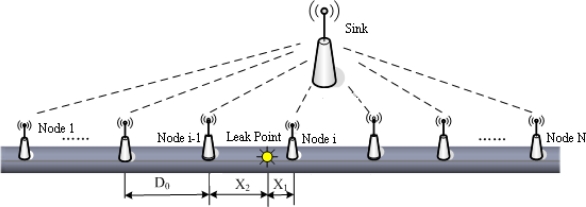
Multi-node integrated localization.

Related research shows that with the increase of the transmission distance, the energy of leak signals will gradually decline, which reflects in the decrease of average amplitude of the signal waveform. Suppose there are *N* sensor nodes near the leak point detecting the abnormal signals when the leak happens. The distances between neighboring nodes are all *D*_0_, and the distance between leak point and two nearest nodes from both sides are *X*_1_ and *X*_2_, respectively (shown in Figure 5). All the sensor nodes, which detect the leak signals, send single mode signals, their own ID and location to the sink.

Suppose *X*_1_ < *X*_2_, the characteristic function method is used to calculate the RMS amplitude of each signal, and the Equation is:

(15) $$V=\sqrt{\frac{1}{\Delta T}\int_{0}^{\Delta T}V^{2}(t)\mathrm{dt}}$$

where Δ*T* denotes signal sampling time. We compare all the signals’ average amplitude *V_i_* (*i* = 1,2,…,*N*) and obtain that:

(16) $$V_{i}>V_{i-1}>V_{i+1}>V_{i-2}>\cdots >V_{1}$$

As can be seen from Figure 5, node *i* is the closest to the leak point, so its amplitude *V_i_* is the largest. In contrast, node 1 is the farthest to the leak point, so the amplitude *V*_1_ is the smallest. Then the signals above are divided into two groups according to an interval grouping criterion. One group contains the signals collected by sensor nodes on the left of the leak point, whereas the other includes the signals collected by nodes on the right of the leak point. That is:

(17) $$U=\{S_{i-1},S_{i-2},\cdots ,S_{1}\},\;\;\;\;\;W=\{S_{i},S_{i+1},\cdots ,S_{N}\}$$

where *S_i_* (*i* = 1,2,…,*N*) denotes the signal sent to the sink by node *i*. If *N_U_* and *N_W_* respectively denote the signal numbers in group *U* and *W*, the number of signal pairs can be obtained from the following Equation:

(18) $$N_{P}=N_{U}\cdot N_{W}=(i-1)\times (N-i+1)$$

##### (2) Determination of Time Difference

The original leak signal is generally a sort of continuity signal, so it is definitely difficult to obtain the exact time when nodes receive the leak signals. In this situation, we adopt cross correlation analysis for signals to get the time difference of the signal arriving at upstream and downstream nodes.

Suppose cross correlation function *R_AB_*(*τ*) between wave *A*(*t*) and wave *B*(*t*+*τ*) with delay time *τ* is expressed as follows:

(19) $$R_{\mathrm{AB}}(\tau )=\frac{1}{T}\int_{0}^{t}A(t)B(t+\tau )\mathrm{dt}$$

where *T* denotes the finite time interval. It will be seen from the Equation (19) that if *τ* is a variable, *R_AB_*(*τ*) is a function of *τ*.

Divide *A*(*t*) and *B*(*t*) into *n* equal time units separately. Suppose *t* = *t_i_*, *A*(*t*) = *a_i_*, *B*(*t*) = *b_i_*, *i* = 0,1,2,…,*n*. If *B*(*t*) has a time delay *τ′* relative to *A*(*t*):

(20) $$\{\begin{array}{ll} R_{\mathrm{AB}}(\tau_{j})=\sum_{i=0}^{n}\;a_{i+j}b_{j}, & j=1,2,\ldots ,n\\ R_{\mathrm{AB}}(\tau_{j})=\sum_{i=0}^{n}\;a_{i}b_{i-j}, & j=-1,\;-2,\;\ldots ,-n\\ R_{\mathrm{AB}}(\tau_{j})=\sum_{i=0}^{n}\;a_{i}b_{i}, & j=0 \end{array}$$

According to Equation (19), *R_AB_*(*τ*) is an integral in a limited time range. In the practical application, data sampling only utilizes the limited part of each wave while wave amplitude of unused part is set to 0. Namely, if *i* > *n*, *a_i_* = *b_i_* = 0; if *j* > 0 and *i* + *j* > *n*, *a*_*i*+*j*_ = 0; if *j* < 0 and *i* − *j* > *n*, *b*_*i*−*j*_ = 0. As a consequence, when |*j*| increases, the number of items in (19) becomes increasingly less and the amplitude of *R_AB_*(*τ_j_*) declines gradually. Eventually, when |*j*| > *n*, *a*_*i*+*j*_ = *b*_*i*−*j*_ = 0 and *R_AB_*(*τ_j_*) = 0. When *τ_j_* = *τ′*, *R_AB_*(*τ_j_*) arrives at the maximum as a result of the same phase between *A* and *B*. Thus we obtain the delay time at the peak point of *R_AB_*(*τ_j_*).

For any functions *A*(*t*) and *B*(*t*) with a delay time *τ_j_*, the cross correlation function *R_AB_*(*τ*) of *A*(*t*) and *B*(*t*+*τ′*) includes the maximum value at *τ_j_* = *τ′*, so this kind of method is applicable to localization of continuous leak signals. For instance, node *A* receives the signal wave *A*(*t*) whereas node *B* receives the wave *B*(*t*+*τ′*) with a delay time *τ′* relative to *A*(*t*), hence leak signal arrival time difference from the leak point to upstream and downstream nodes could be acquired at the peak of *R_AB_*(*τ*). Namely, Δ*t*_AB_ = *τ′*.

Equation (14) is used to calculate the position coordinates of leak point according to Δ*t*_AB_, where wave velocity *α* is also an important parameter. Practically, the spread velocity of the signal wave is affected by many complicated factors, so *α* is determined by actual measurement in the experiment.

##### (3) Weighted Average Localization

All the single mode signals are divided into *N_P_* pairs by signal grouping algorithm, which can generate *N_P_* coordinates according to cross correlation time difference localization method. However, it may generate errors during the process of signal processing. Therefore *N_P_* kinds of localization results are not quite similar. In order to minimize the interference of errors and improve the localization accuracy, weighted average localization algorithm is introduced to work out position coordinates of leak point. The localization Equation is shown as follows:

(21) $$P_{\mathrm{leak}}=\sum_{j=1}^{N_{P}}\;\mu_{j}\;P_{j}\;\;\;\;\;\;(1\leq j\leq N_{c})$$

where *P_leak_* represents the final position coordinate of leak point. *P_j_* denotes position coordinate of leak point calculated by signals in pair *j*. *μ_j_* is the weight factor, which signifies credibility of leak point position determined by signals in pair *j*.

It can be seen from signal attenuation theory and network deployment mode that the farther the distance between two sensor nodes is, the greater the attenuation of received signal is, which results in the decline of *μ_j_*. Therefore, *μ_j_* is inversely proportional to the distance between two sensor nodes. To maintain the precision of final position of leak point, the sum of *μ_j_* (1 ≤ *j* ≤ *N_P_*) should be 1. Hence the Equation of weight factor *μ_j_* is:

(22) $$\{\begin{array}{l} \mu_{j}=\frac{\mathrm{Con}}{L_{j}}\\ \sum_{j=1}^{N_{P}}\;\mu_{j}=1 \end{array}\;\;\;\;(1\leq j\leq N_{c})$$

where *L_j_* denotes the distance between two nodes in pair *j*. And *Con* is a constant, which is used to assure that the sum of *μ_j_* is 1. When values of *N_P_* and *L_j_* are certain, *Con* could be acquired from the following Equation:

(23) $$\mathrm{Con}=\frac{1}{\sum_{j=1}^{N_{P}}\;\frac{1}{L_{j}}}$$

## 5. Simulation Experiments

### 5.1. Experimental Facilities

The outside diameter of the pipeline is 160 mm, the wall thickness of the pipeline is 6 mm and the length of the pipeline is 25 m. The pipeline is filled with a high pressure gas and the pressure is 0.2 Mpa. The leak process is simulated by a leak valve. There are five nodes with the same spacing placed near the leak point. The coordinates of nodes and leak point are 0, 5 m, 10 m, 15 m, 20 m and 12 m, respectively. To make it simple, all nodes are equipped with acoustic sensors. We set the sampling number at 2,048 during a sampling process, sampling frequency is 1 MHz, sampling instant is 2.048 × 10^−6^ s and wave velocity is 4,870 m/s.

### 5.2. Result of Leak Detection Experiment

**Figure 6.**
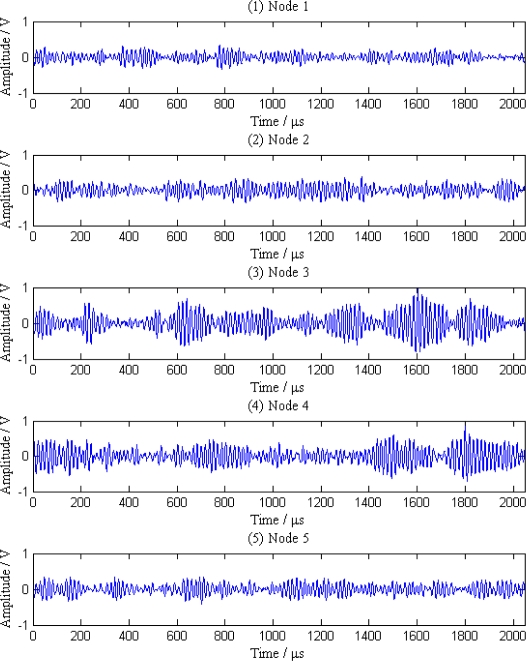
Original signals received by five sensor nodes.

When a leak happens along the pipeline, signal waves received by the five sensor nodes are shown in Figure 6. As can be seen from the Figure 6, the original signals are continuous acoustic emission signals mixed with a great deal of noise and other interference, so it is difficult to distinguish the leak characteristics from these signals.

**Figure 7.**
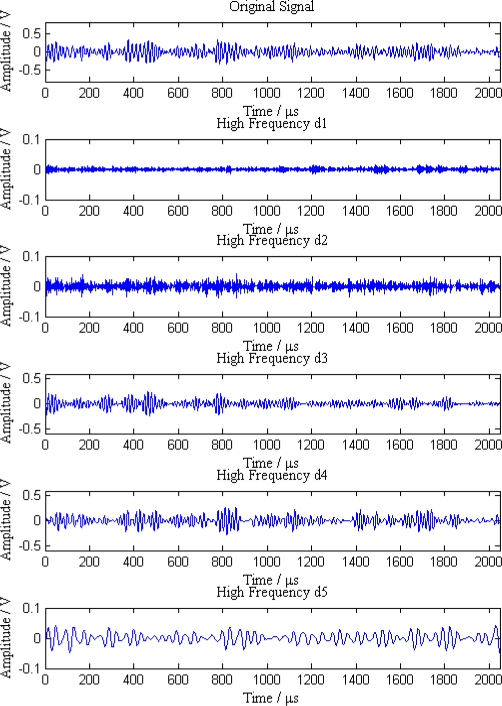
Signal decomposition of node 1.

**Figure 8.**
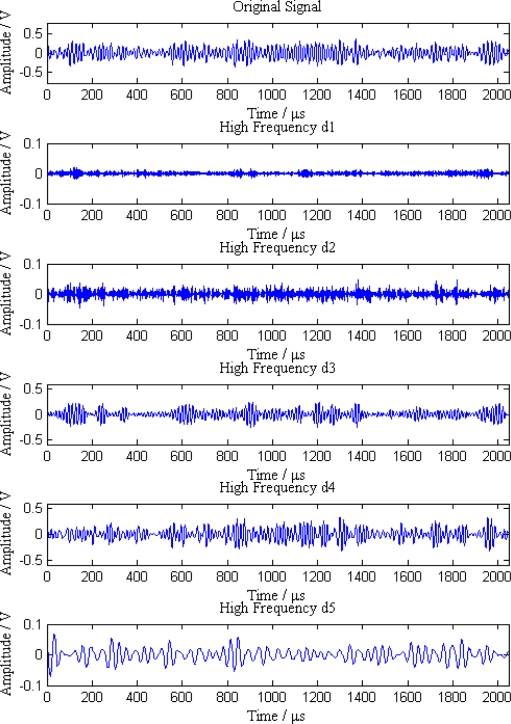
Signal decomposition of node 2.

**Figure 9.**
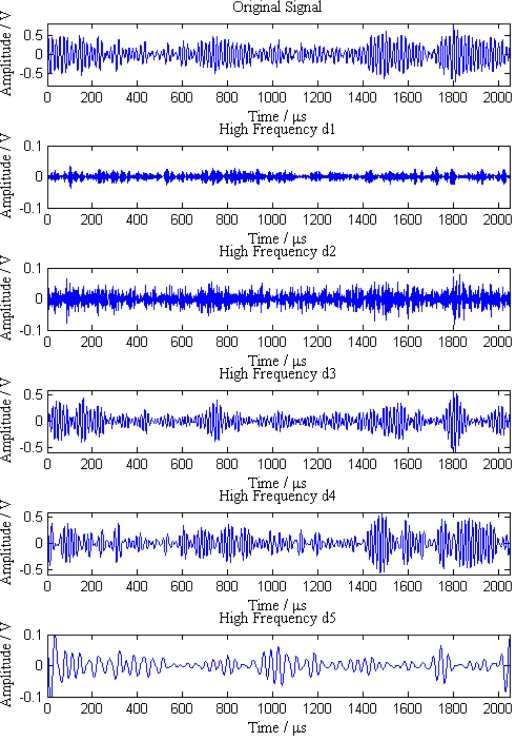
Signal decomposition of node 3.

**Figure 10.**
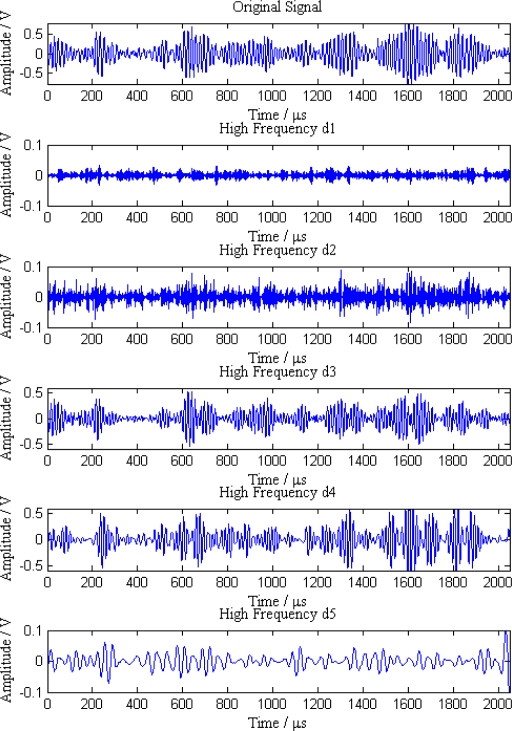
Signal decomposition of node 4.

**Figure 11.**
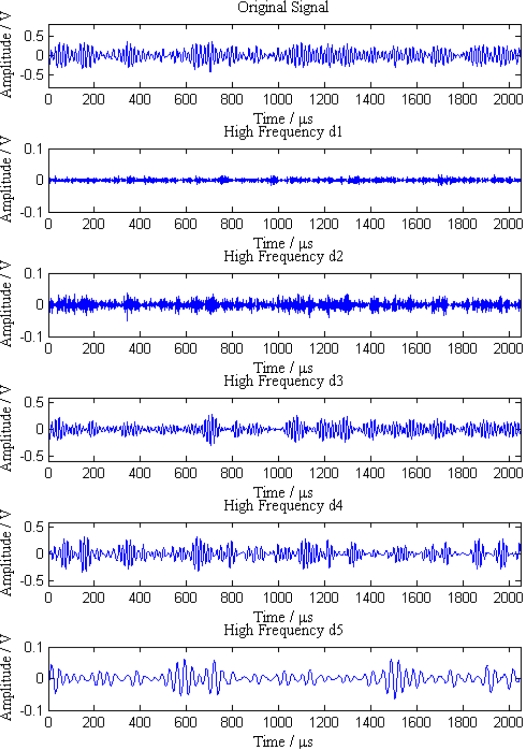
Signal decomposition of node 5.

**Figure 12.**
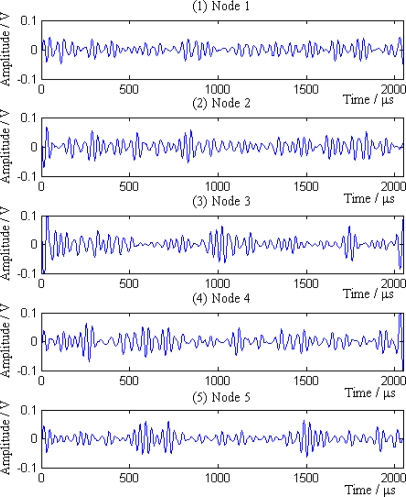
Single mode signals extracted from five original signals.

Additionally, the average amplitudes of original signals obviously decline with the increasing distances between sensor nodes and the leak point. In order to further analyze the leak signals, we use multi-scale wavelet transform algorithm to divide the original signals into five levels. The results are shown in Figures 7–11, where we can see that the signals in the fourth and fifth levels have obvious periodical variation, which can reflect leak characteristics. The signals in the fifth level contain the most energy of the original signals. Therefore, we take the high frequency signals in the fifth level as single mode signals to extract leak characteristic parameters. The results of five original signals dealt with by wavelet transform are shown in Figure 12. Each signal waveform in Figure 12 corresponds to high frequency signal in the fifth level in Figures 7–11. Consequently, the operation above fulfills the preprocessing of original signals, which includes decomposing the signals in multi-scale, eliminating the effect by background noise and extracting the signal ingredients with leak characteristics for further data processing.

Then, characteristic parameters are calculated according to the single mode signals. As characteristic input vectors, they are sent to SVM multiple classifiers for initial recognition. In this paper, 120 groups of data are selected independently as the sample set, which contains 40 groups of large leak state sample, 40 groups of small leak state sample and 40 groups of normal state sample. Among the sample set, 20 groups of each type of pipeline state (60 groups in total) are randomly chosen as the training sample set and the others make up the testing sample set. Table 1 gives the signal examples of three sorts of states: groups 1 and 2 are signals in normal state; groups 3 and 4 are signals in small leak state; groups 5 and 6 are signals in large leak state. We use SVM toolbox in Matlab to train classifiers. Then test samples are sent to multiple classifiers for pipeline state recognition. Output results are normalized finally.

When there is a small leak happening along the pipeline, basic probability assignments determined by SVM multiple classifiers as the initial recognition results are shown in Table 2. From Table 2, uncertainty degrees in leak detection become increasingly large with the increase of distances between sensor nodes and leak point. In addition, the initial recognition result of Node 4 disagrees with that of others so that it is difficult to make a decision according to Equation (13). There are two factors contributing to the phenomenon. One is that sensor nodes have self problems which may lead to the error in characteristic input vectors of SVM multiple classifiers. The other is that the number of samples is so small that the samples could not include all the possible situations and this can cause recognition error of classifiers. To resolve evidence conflict problems, we use an improved evidence combination rule for the final decision.

It can be seen from Table 3 that after evidence combination, the degree of support of proposition *A*_2_ rises to 0.4638. Comparing with the result of single node, the support degree has increased by 37%. In the meantime, the uncertainty degree declines to 0.044, which meets the requirement of Equation (13). Therefore, the diagnosis result supports proposition *A*_2_, which is “small leak”.

### 5.3. Result of Leak Point Localization Experiment

In this part, we use signal mode signals extracted from the original leak signals as the processing objects. According to the process of leak point localization algorithm in Section 4.2, five sensor nodes send their signal preprocessing results to the sink.

**Figure 13.**
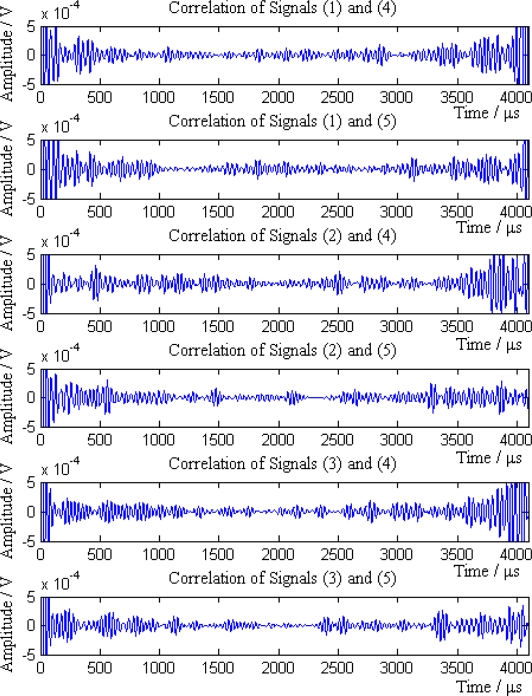
Correlation result of each pair of signals.

Then the sink compares the average amplitudes of single mode signals and divides the signals into two groups *S*_1_ and *S*_2_. *S*_1_ = {1,2,3} and *S*_2_ = {4,5}, where signals in group *S*_1_ come from the sensor nodes on the left of the leak point, while signals in group *S*_2_ are from the sensor nodes on the right of the leak point. According to the Equation (18), *N_P_* = 6. The six pairs of signals are (1,4), (1,5), (2,4), (2,5), (3,4) and (3,5), respectively. The cross-correlation results of above signal pairs are shown in Figure 13.

It can be seen from the Figure 13 that the cross-correlation function of each pair of signals has obvious maximum value. According to the cross-correlation TDOA principle, the position and error of leak point calculated by each pair of signal is shown in Table 4.

In light of the node distance and the value of *N_P_*, we obtain *μ*_1_ = 0.11, *μ*_2_ = 0.09, *μ*_3_ = 0.17, *μ*_4_ = 0.11, *μ*_5_ = 0.34 and *μ*_6_ = 0.17. After weight average combination, the final coordinate of the leak point is:

(24) $$P_{\mathrm{leak}}=\sum_{j=1}^{N_{e}}\;\mu_{1}\cdot P_{j}=11.79$$

Accordingly, the absolute error is 0.21 m and the relative error is 1.75%. It is thus clear that weighted average localization method can effectively enhance position accuracy.

## 6. Conclusions

In this paper we study the features of leak monitoring information and the structure of pipeline monitoring sensor networks, and present a type of pipeline leak detection and localization method based on a hierarchical model. At sensor nodes, to remove the noise disturbance and dispersion phenomena, wavelet transformation technology is first used to decompose the original leak signals, obtaining single mode signals and characteristic leak parameters. Then we set up a SVM multiple classifiers model for the initial recognition of the pipeline state. To enhance the classification effect and avoid a slow convergence rate, a bilinear search method is utilized to select optimal model parameters, which is of great importance for the recognition result. Moreover, a sigmoid function converts the initial recognition results to probability values, achieving the objectivation of the BPA function. At the sink, an improved evidence combination rule is designed for making final decision according to the recognition results from different nodes. The new rule can tackle the evidence conflict problem and increase the degree of focus of the correct proposition. To get the accurate position of a leak point, a weighted average localization algorithm based on cross-correlation TDOA is introduced. Through weighted combination of localization results of multiple groups of nodes, the position accuracy of the leak point is dramatically improved. Experimental results show that hierarchical method can obviously increase the accuracy of pipeline leak recognition (the focal degree of the right proposition has increased from 0.2344 to 0.4638) and avoid the problem of difficult decision making. Additionally, weighted average localization remarkably reduces the process error which is beneficial to localization accuracy (the maximum relative error has decreased from 4.75% to 1.75%). In this paper, we mainly focus on the linear pipeline without considering other pipeline cases. Hence in the future, we plan to carry out a large amount of research on leak detection and localization approaches of pipelines with special structures such as zig-zag or bent pipelines. Besides, we will do a number of experiments to exploit the feasibility and validity of our approach used in pipelines of other materials such as water and oil as well as underground pipelines, finally extending our method to industrial applications.

## Figures and Tables

**Table 1.** Signal sample.

**Peak value**	**Average amplitude**	**Variance**	**Root amplitude**	**Peak factor**	**Pulse factor**	**Margin factor**	**Skewness factor**	**Kurtosis factor**
0.04729	0.01272	0.00025	0.01077	2.96782	0.41931	3.71730	−1.4047	2.69521
0.04496	0.01395	0.00029	0.01195	2.62446	0.38066	3.22298	−1.4020	2.53662
0.07940	0.01381	0.00033	0.01126	4.33524	0.67553	5.74708	−1.3712	3.26362
0.07187	0.01295	0.00028	0.01077	4.28256	0.63152	5.54897	−1.4185	3.13221
0.11165	0.01607	0.00046	0.01320	5.17711	0.88072	6.94743	−1.2839	3.71906
0.12724	0.01649	0.00055	0.01307	5.41933	0.99074	7.71438	−1.5390	4.92900

**Table 2.** Initial recognition result by SVM multiple classifiers.

**BPA**	***A*_1_**	***A*_2_**	***A*_3_**	***Θ***
*m*_1_	0.2614	0.3400	0.2614	0.1372
*m*_2_	0.2465	0.3210	0.2465	0.1860
*m*_3_	0.2397	0.3118	0.2397	0.2088
*m*_4_	0.2344	0.2344	0.3047	0.2265
*m*_5_	0.2279	0.2964	0.2279	0.2478

**Table 3.** Result of decision making.

BPA	*A*_1_	*A*_2_	*A*_3_	*Θ*
*m*_1,2_	0.2742	0.4040	0.2742	0.0476
*m*_1,2,3_	0.2667	0.4469	0.2667	0.0197
*m*_1,2,3,4_	0.2601	0.4296	0.3012	0.0091
*m*_1,2,3,4,5_	0.2467	0.4638	0.2851	0.0044

**Table 4.** Localization result and error of each pair of signals.

**Signal pair**	**(1,4)**	**(1,5)**	**(2,4)**	**(2,5)**	**(3,4)**	**(3,5)**
Node distance (m)	15	20	10	15	5	10
Δ*t* (μs)	1,672	638	684	378	346	996
*X* (m)	3.43	8.45	3.33	6.58	1.63	2.57
Coordinate of leak point *P* (m)	11.57	11.55	11.67	11.58	11.66	12.57
Absolute error (m)	0.43	0.45	0.33	0.42	0.34	0.57
Relative error	3.58%	3.75%	2.75%	3.50%	2.83%	4.75%
